# INTERIM ANALYSIS: VALIDATION OF THE AGITATION IN ALZHEIMER’S SCREENER FOR CAREGIVERS (AASC®)

**DOI:** 10.1093/geroni/igae098.4228

**Published:** 2024-12-31

**Authors:** W Clay Jackson, Jared Stroud, Malaak Brubaker, Mehul Patel, Sheri Fehnel, Sue Peschin, George Grossberg, Carolyn Clevenger

**Affiliations:** University of Tennessee College of Medicine, Memphis, Tennessee, United States; OhioHealth Physician Group, Columbus, Ohio, United States; Otsuka Pharmaceutical, Princeton, New Jersey, United States; Otsuka Pharmaceutical, Princeton, New Jersey, United States; RTI Health Solutions, Research Triangle Park, North Carolina, United States; Alliance for Aging Research, Washington, District of Columbia, United States; St. Louis University, St. Louis, Missouri, United States; Emory University, Atlanta, Georgia, United States

## Abstract

Agitation in Alzheimer’s dementia often goes unrecognized or underreported, contributing to delayed diagnosis and treatment. The International Psychogeriatric Association (IPA) set a standard for defining agitation in cognitive disorders. The Agitation in Alzheimer’s Screener for Caregivers (AASC®), the first clinical tool based on IPA criteria, was developed to support caregiver-healthcare professional (HCP) discussion and timely recognition of agitation in Alzheimer’s dementia. It has 2 questions assessing presence and impact of agitation behaviors. This ongoing, prospective, multisite, single-visit observational study will calculate predictive metrics for recognizing agitation by the AASC® versus IPA criteria. Interim (n=50 caregiver-HCP dyads) and final (n=150 dyads) analyses will determine agreement rates (yes/no) and sensitivity/specificity. This ongoing interim analysis included 44 dyads (caregivers completed AASC®; HCPs completed IPA criteria). Most patients/caregivers were female (70%/50%) and 74 (±7.3)/58 (±18.9) years old. Most caregivers were White (57%), a spouse/partner (43%) or child (23%), and spent a mean (SD) of 40.8 (44.3) hours/week providing care. Percentage agreement between AASC® and IPA was 70.45%; Cohen’s kappa coefficient (level of agreement) was 0.35 (95% CI: 0.08, 0.62). The number of false positives/negatives using AASC® was 3/10; positive/negative predictive values (95% CI) were 0.727 (0.464, 0.991)/0.697 (0.540, 0.854). The strongest contributor to false negatives on the AASC® was caregivers’ minimization of the impact of agitation behaviors on patient functioning, underscoring a key educational gap. These results suggest that the AASC® can improve caregiver-HCP communication and awareness of agitation behaviors and their impact, thereby advancing early recognition of agitation in Alzheimer’s dementia.

